# Carbon (CI) and energy intensity (EI) dataset for retail stores

**DOI:** 10.1016/j.dib.2018.10.080

**Published:** 2018-11-02

**Authors:** Ana Ferreira, Manuel Duarte Pinheiro, Jorge de Brito, Ricardo Mateus

**Affiliations:** a CERIS, Instituto Superior Técnico, Universidade de Lisboa, Av. Rovisco Pais, 1, 1049-001 Lisboa, Portugal; b CTAC, University of Minho, Department of Civil Engineering, Campus de Azurém, 4800-058 Guimarães, Portugal

## Abstract

This data article presents data collected from the 250 highest revenue retailers around the world, assessed according to publicly available data from the fiscal year 2016, in order to determine retailer׳s overall carbon intensity (CI) and energy intensity (EI). Data collection included additional variables such as retailers’ revenue rank, operational typology, number of stores, store sales area and number of workers. Based on this dataset, CI and EI benchmarks were calculated for food and non-food retailers, applying the statistic function first quartile (Q1) for the best practice, second (Q2) and third (Q3) quartiles for conventional practice and fourth quartile (Q4) for worst practice and correlations were tested between the variables "EI", "CI" and "retailer revenue", applying the statistic function CORREL (Ferreira et al., In press) [1]. Finally, a cluster analysis was performed for food and non-food retailers, to identify possible segmentation patterns between the variables “EI”, “CI” and “retailer revenue”. The information provided in this data article is useful for furthering research developments on the influence of isolated variables on retail EI and CI and in assisting retailers, architects, engineers, and policy makers in establishing optimal energy performance goals for the design and operation of retail stores. For further data interpretation and discussion, see the article “Combined carbon and energy intensity benchmarks for sustainable retail stores” (Ferreira et al., In press), of the same authors.

**Specifications table**

**Table:** 

Subject area	*Energy and Environment*
More specific subject area	*Energy and Carbon intensity*
Type of data	*Tables*
How data was acquired	*Online desk research, using software Microsoft Edge HTML 17.17134. For the statistic treatment of the data, software Microsoft Excel for Office 365 MSO was used.*
Data format	*Raw, analyzed*
Experimental factors	*To verify the relation between CI, EI and retailer revenue, data was collected from sustainability/CSR reports of the top 250 highest revenue retailers.*
Experimental features	*A regional analysis of the provenance of retailers was conducted, as well as a segmentation analysis of the way retailers presented EI and CI data. EI and CI benchmarks were calculated for food and non-food retailers, applying the statistic function first quartile (Q1) for the best practice, second (Q2) and third (Q3) quartiles for conventional practice and fourth quartile (Q4) for worst practice. Correlations were tested between the variables "EI", "CI" and "retailer revenue", applying the statistic function CORREL. In addition, a cluster analysis was performed for food and non-food retailers, to identify possible segmentation patterns between the variables “EI”, “CI” and “retailer revenue”.*
Data source location	*Instituto Superior Técnico, Universidade de Lisboa, Av. Rovisco Pais, 1, 1049-001 Lisboa, Portugal*
Data accessibility	*Data is with this article and in the public repository “Mendley data” under the data identification reference: Santos Ferreira, Ana Sofia (2018), “Combined carbon and energy intensity benchmarks for sustainable retail stores”, Mendeley Data, v1*http://dx.doi.org/10.17632/ww29xrsv56.1
Related research article	A. Ferreira, M.D. Pinheiro, J. de Brito, R. Mateus, Combined carbon and energy intensity benchmarks for sustainable retail stores, In press. (2018) [2].

**Value of the data**

• Data can be of use to both international or local retailers in terms of energy and GHG emissions’ management.
• It can be of use to architects and engineers when designing new or refurbished retail, providing key indicators for the design or operation performance of retail stores in terms of EI and CI.
• It may also be of interest for green building tools, as EI and CI “best practice” threshold values, coupled with life-cycle assessments, are also fundamental to perform sustainability assessments.
• Benchmarks may be used for other researchers, as further investigation is needed to identify the influence of isolated variables on retail EI and CI, like location, retail sub-type, energy source, building size or building technologies.

## 1 Data

Supplementary Table 1 ranks the 250 highest revenue retailers around the world. In this table, data collected from each retailer was compiled according to publicly available data [2], under the categories: “Dominant operational category”, “Store typology”, “Number of countries of operation” and “FY2015 retail revenue (US$M)”. Additional data was gathered from retailers’ sustainability/CSR reports [3], [4], [5], [6], [7], [8], [9], [10], [11], [12], [13], [14], [15], [16], [17], [18], [19], [20], [21], [22], [23], [24], [25], [26], [27], [28], [29], [30], [31], [32], [33], [34], [35], [36], [37], [38], [39], [40], [41], [42], [43], [44], [45], [46], [47], [48], [49], [50], [51] for the categories “Number of stores”, “Average store sales area in m²”, “Total store sales area in m²”, “Energy intensity in kWh/m²/y”, “Carbon Intensity in kgCO_2_eq/m²/y”, “Average number of workers per store”, “Total number of workers” and “Revenue per store sales area in $/m²/y”. Based on this dataset, benchmarks for “best”, conventional” and “worst” practice in terms of EI and CI were established, as well as correlations tested between the variables "EI", "CI" and "retailer revenue".

Supplementary Tables 2–7 present the cluster analysis’ segmentation output performed for the variables “CI”, “EI” and “retailer revenue” for food and non-food retailers.

## 2 Experimental design, materials and methods

A qualitative comparison was made on the energy intensity (EI) and carbon intensity (CI) patterns of retail stores, according to the methodology described by Ferreira et al. (2018) [1].

Data retrieved from retailers was organized in a table that included the variables “Dominant operational category”, “Store typology”, “Number of countries of operation”, “FY2015 retail revenue (US$M)”, “Number of stores”, “Average store sales area in m²”, “Total store sales area in m²”, “Energy intensity in kWh/m²/y”, “Carbon Intensity in kgCO_2_eq/m²/y”, “Average number of workers per store”, “Total number of workers” and “Revenue per store sales area in $/m²/y” (Table 1). EI and CI of each retailer were analysed or calculated, normalizing total energy consumption/GHG emissions by total sales floor area per year.

Based on this dataset, CI and EI benchmarks were calculated for food and non-food retailers applying the statistical operation median for “conventional practice” and first quartile (Q1) for “best practice”. The “best practice” was defined as the upper limit of the first quartile, corresponding to the boundary of the 25% lowest values. The “worst practice” was defined as the lower limit of the third quartile (Q3), corresponding to the boundary of the 75% highest values (Ferreira et al., In press) [1]. Additionally, correlations were tested between the variables "CI", "EI" and "retailer revenue" for food and non-food retailers, applying the statistic function CORREL.

Cluster analysis were performed for food and non-food retailers to identify possible segmentation patterns between the variables “EI”, “CI” and “retailer revenue”. The cluster analysis was based in the Sum of the Squared Differences (SSE) between each observation and its group׳s mean (non-hierarchical or k-means cluster analysis). The number of segments was chosen when the SSE of all cases within the cluster dropped significantly, being closer to 0 than with any other segmentation arrangement (Supplementary Tables 2–7).

## Appendix A Supplementary material

Table 1 Dataset of the 250 highest revenue retailers regarding the variables “dominant operational category”, “store typology”, “number of countries of operation”, “FY2015 retail revenue (US$M)”, “number of stores”, “average store sales area in m²”, “total store sales area in m²”, “energy intensity in kWh/m²/y”, “carbon Intensity in kgCO₂eq/m²/y”, “average number of workers per store”, “total number of workers” and “revenue per store sales area in $/m²/y”.

.

Table 2 Cluster analysis’ segmentation output performed for the variables “EI” and “CI” for food retailers.

.

Table 3 Cluster analysis’ segmentation output performed for the variables “EI” and “CI” for non-food retailers.

.

Table 4 Cluster analysis’ segmentation output performed for the variables “EI” and “revenue” for food retailers.

.

Table 5 Cluster analysis’ segmentation output performed for the variables “EI” and “revenue” for non-food retailers.

.

Table 6 Cluster analysis’ segmentation output for the variables “CI” and “revenue” for food retailers.

.

Table 7 Cluster analysis’ segmentation output performed for the variables “CI” and “revenue” for non-food retailers.
